# Team decision-making: the integration of cognitive task analysis and video to accelerate the development of tactical shared mental models in an elite sports team

**DOI:** 10.3389/fspor.2025.1648137

**Published:** 2025-10-21

**Authors:** Pamela Richards, Mandy Robbins, Dave Collins

**Affiliations:** ^1^ School of Health, Social Work and Sport, University of Lancashire, Preston, United Kingdom; ^2^ Department of Psychology, Wrexham University, Wrexham, United Kingdom; ^3^ School of Education and Sport, The University of Edinburgh, Edinburgh, United Kingdom

**Keywords:** naturalistic decision making, reflective practice, tactical decision-making, high-performance teams, performance analysis, teamwork, team intelligence

## Abstract

**Introduction:**

The focus of this article is on developing and operationalising shared mental models in elite sports teams, using a Decision-Making Framework.The Decision-making Framework innovatively integrates Cognitive Task Analysis (CTA) methods into off-field after action review meetings (AAR) to enhance on-field performance.

**Methods:**

Using a mixed method approach, fourteen international players and three expert coaches participated in this study. CTA methods were employed during a 31 day training period, to capture development in individual and team decision-making.

**Results:**

Both qualitative statements and inferential statistics indicate that DM can be accelerated when a slow, deliberate, reflective, off-field digital environment (AAR) is integrated with competitive ‘in-action’, on-field contexts.

**Conclusion:**

The DM Framework accelerated and enhanced individual and team decision-making.

## Introduction

### Naturalistic decision making in sport

One of the key challenges for coaches in sport is developing both individual and team decision-making (DM) skills (1). The dynamic environment of team sports is characterized by high intensity work bouts, involving the integration of motor skills and cognitive function. Such dynamic environments require individuals to perform skills under physical pressure and execute complex, multiple patterns of play in a synchronized fashion (2) to secure success.

DM requires a complex and dynamic integration of several elements and processes, which simultaneously and dynamically interact (3). Such processes include the interaction between situational/tactical aspects (e.g., teammates, opposition, area of the pitch) and strategic factors (team philosophy & match objectives). All of these must be similarly applied by all team members, often without overt in-game communication, through the development and application of Shared Mental Models [SMMs; (4)]. Unfortunately, limited consideration has been directed towards the influence of the playing context in which such decisions are made (5) and the significant role of the player's own subjective, cognitively-based perceptions relating to such situations.

Consequently, practitioners need to take responsibility for understanding and designing training environments that integrate the player's subjective perceptions of situations within the context of the performance setting. Specifically, this paper proposes the integration of Cognitive Task Analysis (CTA) into the learning environment as a method to both assess SMMs, in addition to using CTA as a training approach (4). This paper aims to demonstrate that the integration of CTA can be used to enhance the development of tactical SMMs in sports teams.

### Shared mental models: study and application in applied settings

SMMs schematically represent all the roles and functions of team members involved in a given task (6). Such cognitive representations include a shared understanding of the complete task (represented as the way the team is supposed to perform) and the resources required to complete the task. Each individual's SMM represents a “chunk” of the performance vision, which includes players’ skill sets, team trademarks and benchmarks; all of which enable monitoring of the team's development and performance (1, 2). Each SMM, holding the detailed information relating to cues, data points, teammates, opposition, tactics, all in context of the geographical location of the area of play and related specifically to an aspect of performance (e.g., attacking). As such SMMs shape and define the information which is attended to by team members, facilitating a congruent agreed perception of the situation [Inter-perceptivity, (7)]. Interperceptivity supports both heterogenous accuracy (understanding of knowledge relating to a person's own role within the team) and inter-positional accuracy (understanding the role of teammates, including the task) (8). Enhancing heterogenous accuracy and inter-positional accuracy will result in more effective engagement and teamwork within the shared decision space (9). The common understanding of a SMM amongst performers takes time to develop. However, when on-field learning (usually high speed and dynamic) is combined with off-field, slow, deliberate learning (through a DM Framework using CTA), a shared situational understanding of performance is facilitated, and improvements of team DM are accelerated (4, 10).

The use of such reflective processes within groups has been used in several domains, albeit that terminologies have varied across researchers. For example, Siegel and Schraagen (11) used a process of reflection to make individual's implicit knowledge explicit, facilitating sharing across participants and consequently, more integrated performance. Earlier work by Schippers, et al. (12), had already highlighted the advantage of team reflexivity, most notably in the promotion of innovative practice. Richards (13) facilitated reflection during match time (half time intervals) which focused the reflection on critical incidents which occurred during tactical play, using reflective cards. Additionally, Richards and Ghaye (14) expanded the concept of reflective practice and focused on the reflection of interdisciplinary teamwork in elite sport management teams (creating the concept of “Thinking Teamwork”) with discipline experts (psychologist, strength and conditioner, performance analyst etc.). This research resulted in the construction of a reflective framework to guide team reflection in elite interdisciplinary management teams working in sport [c.f. (14) for Framework]. Most recently, Iedema and colleagues (15) used video as a stimulus for reflexive ethnographies (termed VREs) in medical settings. In all these cases, whilst not referred to as SMMs, the processes used were able to expose implicit reasoning and promote greater team integration and efficacy.

In summary, there seems to be strong support for the use of structured reflexive processes to develop a team's capacity to act in an integrated fashion. Shared thinking, both in terms of structures and reasoning, seems to have been a key underpinning to these performance gains, offering a model for our own work in the hyperdynamic sports context.

### Theoretical application to sport: the role of NDM

Decisions in sport are performed in complex and often unpredictable conditions, under high pressure and with extreme time constraints. Unfortunately, such a real-world approach, heavily laden with contextual information, has often been neglected, perhaps because such information has a bidirectional link between perceptions and action (2). One theory which has gained credence is Naturalistic Decision Making [NDM; (16)]. NDM theories of Recognition Primed Decision Making (17), Situational Awareness [SA, (18) and Sensemaking (16)] make a valuable contribution to enhancing our understanding of how SMMs are developed. Importantly, however, it seems that use of multiple theories from the same NDM paradigm offer the most effective approach in sports settings (19).

For example, RPD (20) proposes a dual system, which integrates intuition and subjective analysis of the situation. In field hockey, for example, at an intuitive level, cues from where an attacker is carrying the ball and the angle of the stick will inform potential moves that the attacking player might take. At a more complex level, these technical cues, combined with identified elements from the tactical environment (Situational Awareness), can inform decisions at an individual level and collectively at a defensive team level (action involving multiple players). In contrast, sole reliance on a cue-only driven approach would often result in DM errors. One common tactical error in sport is defending the immediate option being “shown” by the attacker, but permitting a pass which carries greater threat to the team. Clearly, although RPD makes a valuable contribution, reliance on intuition alone is too risky, as pattern matching can generate flaws in perception (21).

Extending this scope, the three-level hierarchical model of SA (18) allows individuals to predict future situations by integrating past experience into the present situation. Indeed, Caserta and Singer (22) proposed that level 3 SA distinguishes elite from non-elite performers in any domain. Finally, sensemaking, which according to Hansen and Andersen (23), enables individuals to make sense of cues in context of the playing situation and team's philosophy. According to Hansen and Andersen (23), sensemaking requires individuals to engage with the process of “noticing” and “framing” elements, using the process of interpretation to make sense of what is noticed and how it is framed. This ability to frame the context therefore supports the inclusion of RPD and Situational Awareness as part of the continuum where cues in the environment and information are made sense of in context of the playing situation and team's philosophy.

RPD, SA and Sensemaking are important components in making a team's action coordinated; in other words, they account for how a team SMM can operate. Thus, each approach makes a valuable and distinctive contribution individually but, when integrated, they provide a comprehensive justification of the possible mechanisms through which DM skills might be developed in sport (19). In an attempt to address this, Richards, et al. (1), developed an integrated approach of multiple NDM theories/models. A DM Framework connected along a continuum of perceived cues (RPD), in the context of the situation (SA) which are interpreted in a bespoke manner to the performance context (Sensemaking).

Extending work by Klein (6, 24), and reflecting the interactive approaches used by Ledema et al. (15), Schippers et al. (11), and Siegel and Schraagen (25), this paper also argues that both psychomotor and psychosocial components of DM are essential dimensions of a SMM and are required in sport. The DM Framework (1) proposes that team DM requires the complex interaction of psychomotor (technical, e.g., technical execution, cue identification, interpretation of situational information and physical movement etc.) and psychosocial processes (non-technical, e.g., creating of a shared vision and common language amongst coaches and players within the context of shared team philosophy; termed an “Alpha Vision” cf (2). The creation of pedagogical processes that address psychomotor and psychosocial mechanisms are outlined in Models 1 and 2 of the DM Framework, resulting in the effective identification, interpretation and communication of key information in competitive play, leading to successful performance.

The DM Framework (1) was utilized in this investigation to accelerate team DM in an elite netball team, preparing for a world cup. The study highlights how individual cognitive thought processes can be collectively developed in a progressive manner to establish a collective mindset (26) and development of SMMs (27) of performance which can be operationalized on the field of play with the integration of CTA approaches.

### The applied context: developing team DM in elite netball

Like soccer, netball is a fast-action, high-pressurized, dynamic team sport. However, the characteristics of netball produces some additional challenges, which are representative of other domains such as military and emergency services etc. Enabling the research outlined in the paper to be transferred to other naturalistic decision-making domains, where individual and teams make tactical decisions under pressure, for example, military (9) and emergency services (28, 29) facilitating cross domain knowledge sharing. First, the smaller playing area in netball provides less space for movement. This requires the seven players to make sharper movements that require enhanced DM skills (4). The area of a netball court is 35 m long and 15.25 m wide; significantly smaller than football pitches. DM in netball replicates circle-related play in field hockey (or the 18-yard box in soccer), where the play is “compact” owing to multiple players being gathered in a small area of the field (30). Second, the rules of netball only allow players to hold the ball for no more than three seconds, after which a pass must be made. Finally, players cannot run with the ball, in contrast with field hockey and soccer, where it is possible to travel further with the ball, until the right passing channel opens or a player becomes available. The combination of these three additional parameters (court size, the three-second rule, not able to travel with the ball) makes netball a highly pressurized sport and thus, an ideal environment to further examine the DM Framework outlined above with the integration of CTA processes.

To explore the application of the DM Framework with integrated CTA processes a specific aspect of the game was identified, in which tactical SMMs could be developed to enhance performance play; namely, the “*attacking centre pass*” (ACP). This paper examines the development of a SMM to facilitate improved execution of an ACP through the integration of Cognitive Task Analysis (CTA) which is embedded in the observation of digital video clips [DM Framework, cf. (1)]. The challenge is to convert a team's ACP to a goal scoring opportunity. Scoring a goal from your own centre is referred to as a *centre pass completion,* and is a key objective for any team. ACP is a dynamic fast pace tactical action requiring an integration of four to seven players in a confined space, in a duration of less than 5 s.

### Purpose of the study

The purpose of this study was to test the application of the DM Framework (1) to the sport of netball at a high-performance level. The study is unique as it embeds CTA approaches into the naturalistic preparation of an elite team preparing for a world cup. The integration offered by the DM Framework enabled CTA to be used as both a training approach and as a means to explore the development of SMMs in an elite performance context. Through integrating CTA with video, into off-field training, we ensured the dual emphasis on building both technical (psychomotor) and non-technical aspects (psychosocial—communication, empowerment, refection) into the team's SMM.

## Methodology

### Background

The objective of this investigation was to transfer the development of tactical SMMs generated in an off-court, slow deliberate learning environment, through the mechanisms of team after action reviews, into on-court, collective decision-making and rapid actions, in high-pressurized competitive match settings.

The research design spanned a period of two years and involved a DM Framework (1) consisting of five stages to develop team SMMs [cf. (4) for five staged model]. The framework has been empirically tested in a number of sports including field hockey (2), netball, Richards et al. (4), and professional football (10, 31, 32).

### Research design

Employing the concept of combinatorics (integrating several CTA methods simultaneously within one design—(33), a combination of CTA approaches were identified and employed to address the different cognitive processes involved in team DM and to develop SMMs. Most NDM researchers use a combination approach when working within the naturalistic setting to capture multiple cognitive processes (33). Table 1 outlines the innovative approach taken to integrate CTA approaches used (combinatoric) and their application to the development of a tactical SMM relating to the ACP. Within the context of this study, multiple CTA methods were integrated into a DM booklet which was utilized in the off-court learning environment (after action reviews). Therefore, making a unique and original contribution not only to how we develop SMMs, but also advancing methodology in relation to team decision-making.

**Table 1:** Combinatorics: CTA approaches integrated into decision-making booklet and off-field learning context to develop SMM in netball players.

CTA method	Stages of method & description of stage	Integration of stages of CTA: exploring team DM in netball players
Critical decision method (42)	Stage 1 preparation	Development of sheets for players by researcher & coach. Bootstrapping with researcher and coaches
	Stage 2 incident selection—centre pass clips identified by coaching team.	DM booklet, (separate sheet per clip) Left hand side page one required the identification of critical situation.
	Stage 3 incident recall	DM sheet—pages 1 and 2. The player “sweeps” through the incident by recalling and retelling it in a written form in a logical process relating to time (Phases) of the centre pass.
	Stage 4 incident retell	
	Stage 5 time line verification	
	Stage 6 progress to deeper understanding	DM Sheet for clips 3 & 6 for each game required players to outline in more detail (i) their own role in the situation (ii) the role of teammates in the situation. Separate pages in DM booklet.
	Sage 7 “What ifs”	DM Sheet Facilitated the team discussion at each phase of every clip. This facilitated the discussion relating to “what ifs” and the development of SMMs.
Concept mapping (43)	Stage 1 selection of area for focus	DM sheets—Page 1 of each sheet (separate sheet for each clip) required players to outline the identification of the specific attacking centre pass situation.
	Stage 2 arrangement of concepts	DM Sheet for Clips 3 & 6 for each game required players to outline in more detail (i) their own role in the situation (ii) the role of teammates in the situation. These enabled players to schematically draw the situation, illustrating the connection between tactical concepts. Separate pages in DM booklet.
	Stage 3 linking of concepts	DM sheets—Diagram for clips 3 & 6 illustrated how concepts were linked and the associations the players held regarding different components.
	Stage 4 refinement of concept	DM sheets—At the end of each phase the team (players and coaches) would discuss the detail of the clip—outlining tactical concepts and roles of individuals.
	Stage 5 new relationships identified	
	Stage 6 building new knowledge and new MM	DM sheets—At the end of each phase the team (players and coaches) having discussed the details of the clips, discussion would progress to defining future actions required of individual team members and the team collectively.
Think aloud problem solving (43)	Stage 1 talking thoughts	i. DM sheets –the players talk aloud (verbalises) their thoughts relating to the attacking centre pass, this results in the development of Shared common language and SMMs.
	Stage 2 probing questions asked	i. DM sheets—During the meeting players and the coaching team collectively probes the incident the team is reviewing. This results in the refinement of existing MM/ SMMs and the development of new SMMs where required.
	Stage 3 protocol analysis	Not undertaken in this study.

CTA involves a range of techniques concerned with the elicitation of knowledge of experts (33) in a naturalistic setting. Such techniques have proved successful in capturing the unobservable cognitive processes, decisions and judgements embedded in expert performances (34, 35). In addition to the CTA approach being used as a method of knowledge elicitation, CTA processes are integrated into training and educational settings to develop cognitive skills and support the development of instructional designs (4, 17). This study therefore integrated CTA through an innovative approach into everyday practices in the naturalistic elite performance setting.

Design of the DM sheets incorporated the CTA approaches of the Critical Decision Method (exploring cues perceived by the players and a timeline of events), Concept Mapping (allowing elaboration of SMMs of players in a visual format and the relationships between cues) and Think Aloud Problem Solving (establishing SMMs and an agreed common language). The CTA approaches were integrated into off-field learning environments using video relating to competitive training games and international matches. The sheets were completed in team meetings as part of a natural team after action review process.

The team utilized in this study was an international netball team preparing for a World Cup tournament. The sensitivity of this performance setting, combined with the integration of tactical play bespoke to the team, removed the option of using a control group as part of the research design.

### Participants

#### Players

Fourteen elite female netball players, with a mean age of 18.9 years (SD = 0.9) participated in the study. All participants were part of a National Under 21 (U21) team preparing for a World Cup tournament and had experience of playing in junior representative teams for a minimum of one year. All players had participated in performance squads and were playing regular high standard domestic club netball.

#### Coaches

Three elite female netball coaches already working with the national squad participated in this investigation. The coaches were identified as high-performance coaches by their National Association. All coaches had coached for a minimum of five years in a high-performance environment and had experience of working with international players and performance programmes.

### Equipment

To facilitate the viewing of performance clips, Dartfish software TeamPro was used to capture and record match footage. A Mac laptop and projector were used to display the identified clips. A reflective DM booklet was designed to address the development of SMMs relating to the *ACP* which incorporated CTA approaches. Table 1 outlines how CTA approaches were integrated into the DM booklet.

### Procedure

All players and coaches gave informed consent prior to the start of the study. It was agreed (Stage 1 of this study) that the focus of this investigation would be the development of a team SMM relating to the ACP which involved the inclusion of players’ knowledge and experience.

Data were captured in relation to the development of SMMs during seven matches over a 31-day competition period during the World Cup campaign. During this period, two phases of preparation were used. Phase one involved training matches undertaken as part of a residential training camp setting, the training preparation phase (training games, *n* = 4). The second phase involved an international tour, the international preparation phase (International Matches, *n* = 3). During both phases, players engaged with off-court learning environments where SMMs would be designed, shaped and refined and on on-court environments where the SMMs developed were operationalized. Off-court video sessions would be engaged with, the morning after a match which had been played the night before (i.e., viewed within a 24-hour window of playing).

#### Capturing of video clips and clip design

During both training and international match phases, the coaching team would view all the centre pass video clips following the match, and identified six video clips relating to the team's match performance.

Performance indicators (Table 2) were applied to the captured training match footage to identify the six best clips (each averaging 14 s in length) that represented the playing constructs required. Within this study we explore the components of SMMs through the sequential changes of DM and behavioural science (team tactical action) through the analysis of nano codes which cascade from a decision tree of primary, secondary and tertiary performance indicators. The establishment of the decision tree is represented in a “code book” for metrics relating to SMMs assessment. Notational analysis enabled match data to be obtained and analysed relating to team performance. The clips represented three types of tactical strategies. For the six clips identified, three clips related to strategy one, two clips related to strategy two and one clip related to strategy three. Based on findings from Richards et al. (4), strategy one was perceived by the coaching team to be the most important so was identified as the option which was emphasized, with expected transference to other strategies which had been discussed with the team.

**Table 2:** Performance indicators and operational definitions used for notational analysis and statistical analysis.

Performance Indicator	Operational Definition
Number of centres	The attacking centre pass relates to the action to start the game.
P0	Passes made from the starting centre and not secured on the first pass.
P1- pass to centre	Passes made and received in the centre third area of the court immediately following the centre pass.
P2—pass into attacking third	Passes received and secured in the attacking third of the court but outside the circle, following the initial centre pass.
P3—pass into circle	Passes received and secured in the attacking circle.
Centre to shot—shot missed	A passage of play where the ball moves from the first centre pass to the circle and a shot is taken. Possession is maintained by the team who took the centre pass. A goal is not scored.
Centre to goal with rebounds	A passage of play where the ball moves from the first centre pass to the circle and a shot is taken. Possession is maintained by the team who took the centre pass. A goal is scored but after the ball has rebounded from the first (or other attempts) at goal.
World class centre	A passage of play where the ball moves from the first centre pass to the circle and a shot is taken. Possession is maintained by the team who took the centre pass. A goal is scored on the first attempt at goal.

The six clips identified would be edited with a pre and post lag time of 3 s from the start/end of the centre pass. Each clip lasted a duration of approximately 14 s, resulting in a total of only 90–120 s of video being used per after action review session (2, 4). The team would view the video clips in conjunction with completing the DM booklet for each match during training and international phases.

**Figure 1:**
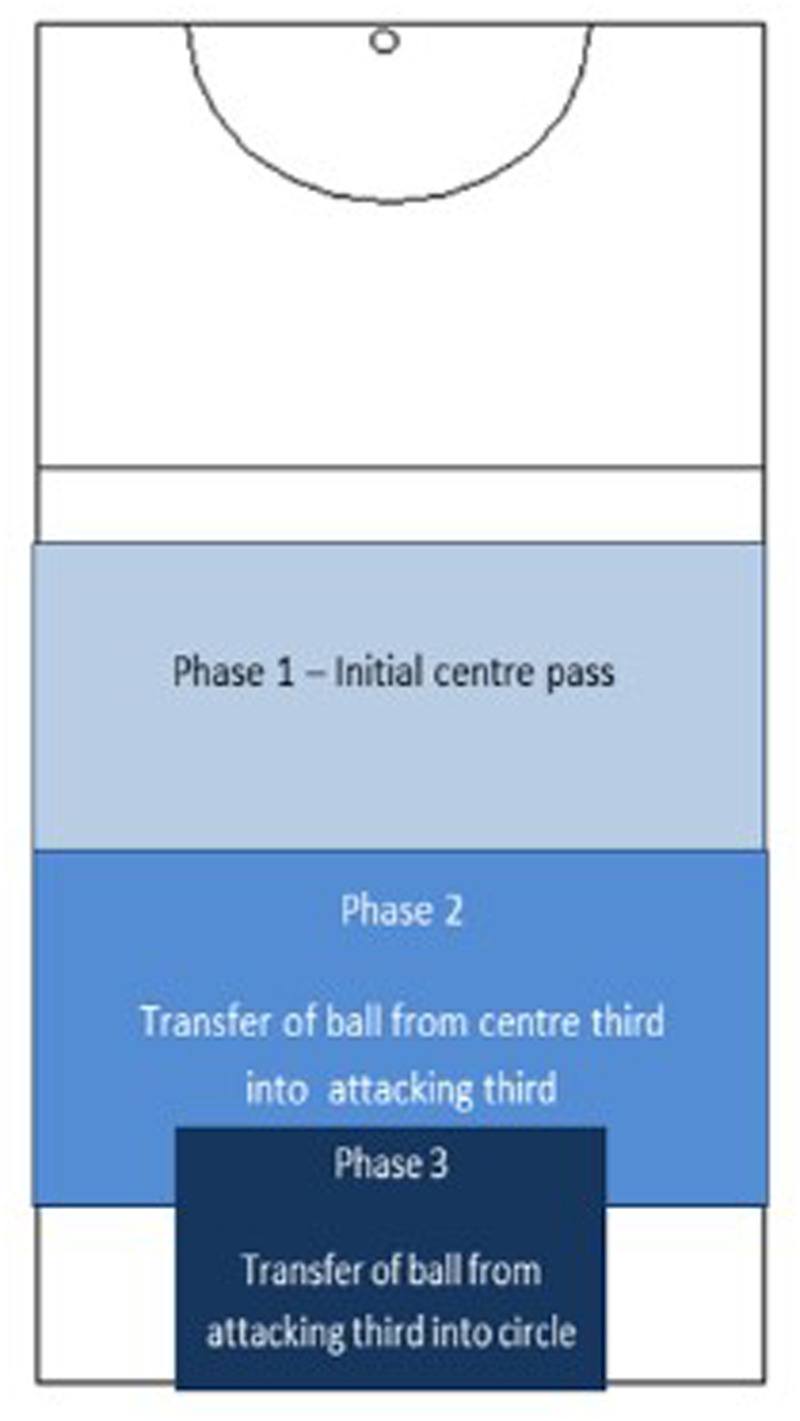
Illustration of netball court and inclusive of phases used in this study relating to an ACP (phase 1, 2 and 3).

Each clip was divided into three phases (Figure 1). Phase 1 represented the initial pass taken to start the centre pass sequence. Phase 2 focused on the ball being transferred from the centre third into the attacking third of the court. The final phase (Phase 3) represented the transfer of the ball from the attacking third into the attacking circle. All clips started with a three second pre-roll and lasted for approximately 10–14 s. This process was replicated for all four matches, enabling six clips to be viewed in post-match after action reviews.

### Integration of the decision-making booklet with the viewing of the clips

Engagement with the DM booklet and the video facilitated the communication of knowledge relating to the ACP. More specifically, the information collected from the reflection on the video and engagement with the DM booklets indicated the following: (i) player's perception of their individual performance; (ii) which technical and/or tactical elements had contributed to their DM, enabling them to execute the particular action; (iii) what information the players attended to at different phases; (iv) location of team mates and opposition at different phases; (v) any interplay between technical and tactical elements; and (vi) the issues that had affected their DM during each incident, including their position and location on the playing court.

**Figure 2:**
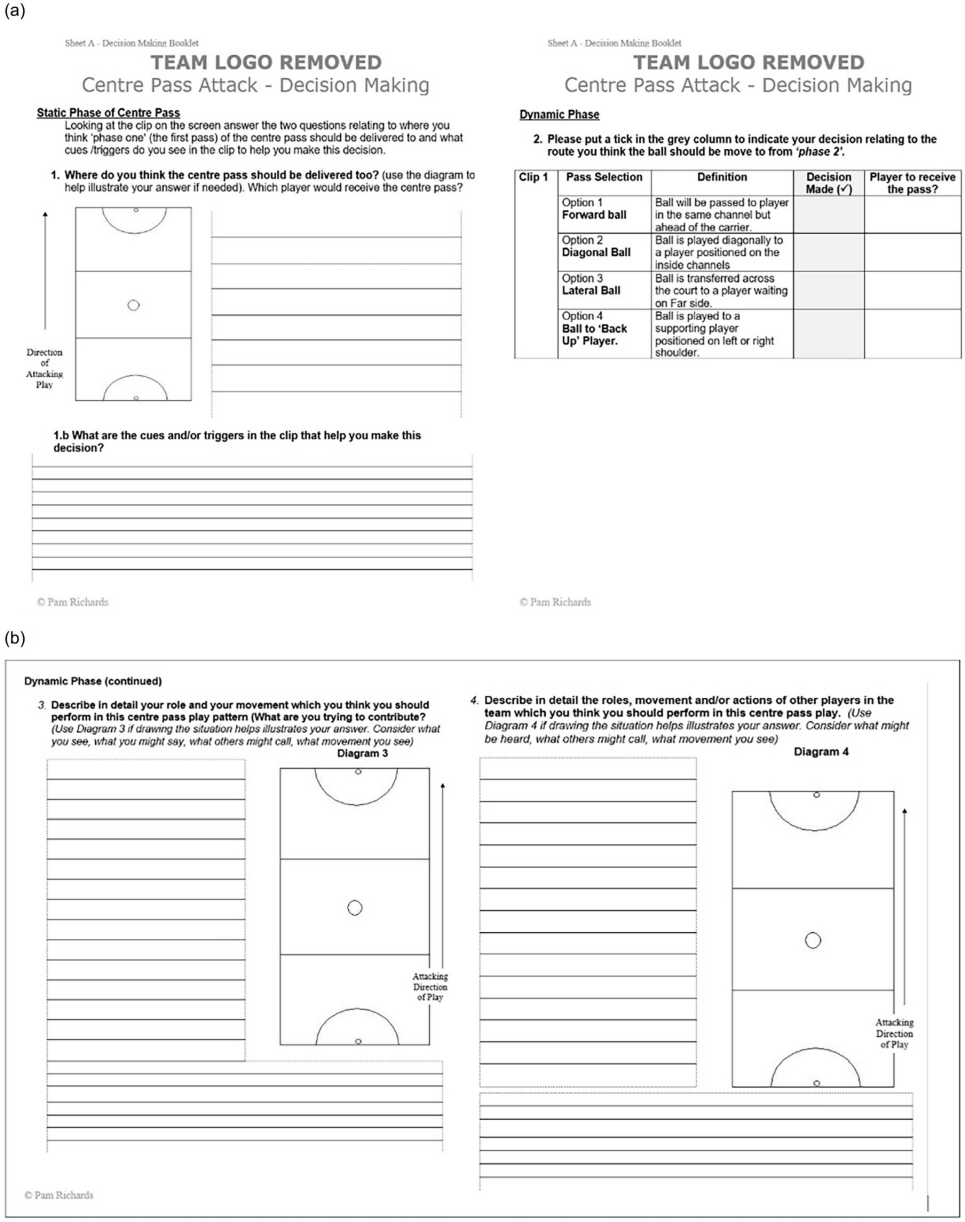
Sheet **(a)** Player reflection sheet—questions 1a and 1b completed for all clips (clips 1–6). Sheet **(b)** Player reflection sheet—role of self and teammates (additional sheet completed by all players for clips 3 & 6).

Players were familiarized with the process of viewing clips in three phases. Players were presented with a DM booklet in team video meetings. The DM booklet consisted of sheet A and sheet B endorsed by the coaching team. Sheet A would be used to review clips 1, 2, 4 and 5 (Figure 2a). Players were required to write their reflection at specific phases of the ACP based on observations and reflections of the video. Question 1 related to phase one (when the centre player was standing with the ball waiting for the whistle), in which the players were asked to reflect on the information and cues in the environment which they needed to be aware of (this matched the team SMMs). Question 2 related to phase two for the centre pass, where players were asked to reflect and make a decision indicating where the most effective pass should be played. For clips 1, 2, 4 and 5 the video was played and stopped at the phases outlined above, and the players wrote their responses in the DM booklet. After the players had written their responses, the clip was played again and the group discussed the clip in relation to cues in the environment, role of the teammates and the relevance of the clip in the context of the team SMMs.

**Figure 3:**
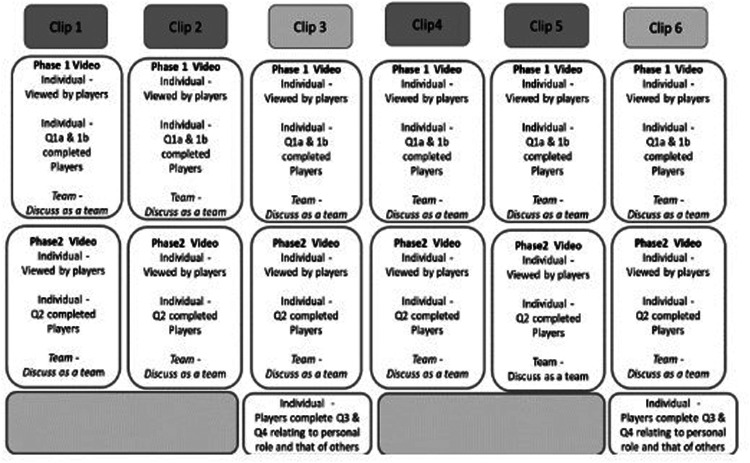
Schematic illustration of how the decision booklets were completed, including the order of questions which were responded to and when team group discussions were held.

Viewing of clips 3 and 6 involved the same process as presented above for Clips 1,2,3 and 5; however, additional information was sought on sheet B for clips 3 and 6 only (see Figures 2b). This required the players to write in detail what their role was in the specific situation against the role of others (incorporating situational awareness & sensemaking). After viewing each clip (clips 3 & 6) the team once again discussed the clips verbally. Figure 3 schematically illustrates how the DM booklets were completed, including the order of questions and when team group discussions were held.

Finally, at the end of the team meeting, players were presented with their statistics from the same match they were reflecting on in the form of team behaviour metrics. This included established on-going statistical feedback (such as turnovers, goals, etc.) in addition to specific statistics relating to the ACP. This process was repeated for each of the four consecutive matches played during holding camp.

### Data processing

CTA analysis was undertaken on all players for all six clips per game for seven games. Coaches’ responses to the DM booklet (for games 1–7) enabled an expert's benchmark response (master vision) to be established, providing the standard against which player responses could be compared.

Data analysis examined the DM sheets at multiple levels. This included all DM booklets per players for all matches (training and international), in addition to the clustering of layers at (1) players connect to specialist playing units within the team (attack, centre & defensive units) and (2) the team as a collective entity. Finally, the data were also analysed, to compare training matches with international performance with regard to responses to (1) CTA data collected through the DM booklet, and also (2) match analysis data through performance statistics. In addition, the analysis examined match data relating to team performance in the competitive setting. Statistical data collected through the mechanism of match analysis also enabled data relating to match performances to be evaluated. Data collected from match statistics were descriptively analysed using quantitative methods to assess the team's performance and its relation to team DM. This multi-level approach allowed for sequential analysis of the DM sheets between training and international matches to be explored, while simultaneously carrying out the analysis of CTA data at an individual player level, unit level and collectively at the team level.

## Results

Data analysis is presented in two parts. Part one presents analysis of data relating to the off-field learning context [After Action Reviews and Debriefs, c.f. (4) for outlined of process], (DM booklets using CTA). Part two presents the transference of off-field learning into the competitive setting.

### Part 1 off-court, slow deliberate learning (DM framework with CTA)

*Phase 1 analysis: Recognition and identification of cues and for Phase 1 of the ACP:* The DM booklet incorporated CTA methods to obtain the cognitive demands for Phases 1, 2 and 3 of the ACP. However, specific emphasis was placed on Phase 1 and the influence which information perceived in this initial stage, had on informing later decision-making in phase 3 (32, 36). Previous work by numerous authors [e.g., Bate & Richards (31), senior soccer; Richards, Penrose and Turner (10), premier league youth soccer] support the claim that application of the DM framework enhances DM at Phase 1 which, in turn, enhances decisions made in the later stage of the action (Phase 3). This section illustrates the themes generated (information attended to) by players and expert coaches for phase 1 of the ACP. Such analysis provided insights on what information players were attending to at the start of the centre pass (Phase 1), and how the processing of such information might help inform the development of SMMs and the team's DM process.

At an individual level, a total of 25 themes were identified by both players and coach, with a further 5 themes being identified by players only for phase 1. These additional themes were: “starting position of teammate”, “multiple options being observed”, “distance”, “timing” and “individual tactical concepts” (e.g., a player being forced wide). Consideration was also given to the analysis of themes relating to environmental cues and data points (influencing factors e.g., score line, time left etc). Themes generated were analysed at an individual level, in addition to being explored within specific playing units (attack, centre court unit & defence) to assess SMM at a sub-unit level. While all this information is of interest, six themes from the 30 themes were identified by each playing unit and the expert coaches. These six themes were all ranked in the order of frequency, for each playing unit and the expert coaches and as a result have been termed “dominant themes”. “Dominant themes” are aspects perceived within the performance setting to be of importance, in influencing and informing decisions which are collectively made. “Dominant themes” are illustrated in Table 3.

**Table 3:** Analysis of ranked order themes identified for phase 1 of the attacking centre pass for playing units, coach and all players (response to question 1b).

Rank order	Higher order theme—ranked order for coach	Higher order theme—ranked order for attacking players (E.g. GS/GA) (highest to lowest)	Higher order theme—ranked order for centre court players (E.g. WA/C/WD) (highest to lowest)	Higher order theme—ranked order for defensive court players (E.g. GD/GK) (highest to lowest)
1	Multiple players recognised and connected to own teammates (e.g., “GA/WA on same side) (* = 3 + players) (*n* = 20)	Multiple players recognised and connected to own teammates (e.g., “GA/WA on same side) (*n* = 20)	Multiple players recognised and connected to own teammates (e.g., “GA/WA on same side (*n* = 38)	Multiple players recognised and connected to own teammates (e.g., “GA/WA on same side) (*n* = 38)
2	Connection between teammate, position (on-line/offline) and location of defender (“GA ahead of opponent” and “online with WA ahead of WD”) (*n* = 16)	Connection between teammate, position (on-line/offline) and location of defender (“GA ahead of opponent” and “online with WA ahead of WD”) (*n* = 16)	Connection between teammate, position (on-line/offline) and location of defender (“GA ahead of opponent” and “online with WA ahead of WD”) (*n* = 24)	Team SMMs (e.g., staggered/“stack'/ “Casey”/“Classic”) (*n* = 29)
3	SMMs Concept + “space”/Ball side and none ball side (e.g., GD is ball side of WD on GA side) (*n* = 15)	Predictive thinking (e.g., ready for second phase—3 rd. phase = *) (*n* = 15)	SMMs Concept + “space”/Ball side and none ball side (e.g., GD is ball side of WD on GA side) (*n* = 20)	Area of Court (e.g., Offline) + player positioning (Online/over the line) (*n* = 20)
4	Area of Court (e.g., Offline) + player positioning (Online/over the line) (*n* = 10)	Team SMMs (e.g., staggered/“stack'/ “Casey”/“Classic”) (*n* = 15)	Players Zone Space (“on their left shoulder) (*n* = 20)	Area of Court (e.g., Attacking Third) (*n* = 14)
5	Team SMMs (e.g., staggered/“stack'/ “Casey”/“Classic”) (*n* = 8)	Teammate Undefended (Free player) (*n* = 13)	Physical movement of teammate (e.g., Speed of teammate/Dodge/angle of run) (*n* = 19)	Players Zone Space (“on their left shoulder) (*n* = 12)
6	Teammate Undefended (Free player) (*n* = 8)	SMMs Concept + “space”/Ball side and none ball side (e.g., GD is ball side of WD on GA side) (*n* = 12)	Area of Court (e.g., Attacking Third) (*n* = 18)	Connection between teammate, position (on-line/offline) and location of defender (“GA ahead of opponent” and “online with WA ahead of WD” (*n* = 9)

Note: Shading of themes illustrate the patterns of similarity of themes across the different playing units, illustrating the similarity across the different specialist units of play. Each theme being assigned to one colour of shading to illustrate the pattern.

Such “dominant themes, influences the collective team decisions which are made. Two of the most important dominant themes were (i) “*multiple players recognized and connected to own teammates*” and (ii) “*connection between teammates, position on-court (online/offline) and location of defender*”. “Both were ranked within the top two themes for two of the playing units (attacking and centre court units) and independently, the coach. In addition, both the defensive and centre court units produced the highest recognition frequency count, with 38 identifications for “*multiple teammate*” specifically, (defensive unit, *n* = 38; centre court unit, *n* = 38). Recall frequency from the coach and the attacking unit players produced the same value of *n* = 20 identifications for the theme, “*multiple players*”.

**Figure 4:**
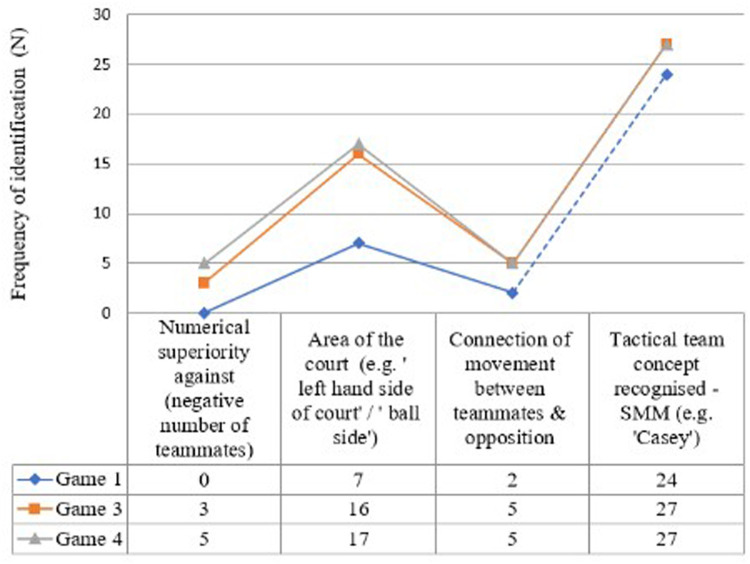
Comparison of four dominant themes identified by players in phase 1 of ACP for game 1, 3 and game 4.

The second theme “*connection between teammates, position on-court and location of defender*” was ranked second in order of recall frequency for the coach (*n* = 16), and attacking unit (*n* = 16), with the highest recall frequency being produced by the centre court unit (*n* = 24). Further descriptive analysis indicated that both key dominant themes of (1) “multiple players recognized and connected to own teammates” and (2) “connection between teammates, position on-court and location of defender” increased in recall frequency for Phase 1 from game 1 to game 3 (Figure 4). The pattern of progression was the same for all three playing units with a rapid increase in identification of recall frequency for these two themes in game 3. All playing units increased their identification of “multiple players” theme over the four match period. Centre court players increase their identification progressively over games 1–3 with recognition values of *n* = 4, *n* = 8 and *n* = 16 respectively. Following the two-week period of non-playing contact all three playing units maintained a higher value of identification than first experienced in game 1, supporting the findings of Richards, et al. (10),. Such findings suggest that players were more able to identify connections between teammates and contextualize this in relation to the opposition within the playing context of Phase 1 of an ACP, therefore suggesting a shared common perception of the performance setting. Such evidence suggests that information relating to “*multiple teammates*” and “*connection between teammates*” are key factor in the decision-making process of the ACP.

Figure 4 illustrates the changes in the “dominant” themes in Phase 1 for games 1, 3, and 4. The development of specific SMMs (e.g., “Casey”, language allocated to a specific type of tactical centre) developed in the first team meeting was immediately recalled and recognized by players to inform DM in Phase 1 of the ACP. Indicating a shared common language. The theme “*tactical team concept* recognized a specific SMM” (indicating specific reference to “Casey”) was the highest ranked theme for all playing units (Figure 4) and was ranked third highest for the coach's recall frequency (Table 3). This is only to be expected, given that the focus of meeting 1 with the players on the first contact point was to develop a SMMs relating to an ACP.

A second point worthy of recognition is the increase in the theme “*numerical superiority*”. This theme was ranked as the priority by the coach for frequency of recall, but was not recognized by any player in game 1. Recognition of this theme increased in game 3 (*n* = 3) and continued to increase to a review point for game 4 (*n* = 5). It is interesting to note that a similar pattern was also identified for increased frequency of recall for the theme relating to “*connectivity between teammates and the opposition*”*,* perhaps suggesting a connection between themes, however more research is required to explore such a connection.

Finally, players perceived “*area of the court*” to be influential in Phase 1, with players’ making (*n* = 7) references to related raw order themes in game 1. The theme of “*area of the court*” rapidly increased in game 3 (*n* = 16). Following the two-week period of non-contact, the theme “area of the court” remained dominant in recall frequency for players.

**Figure 5:**
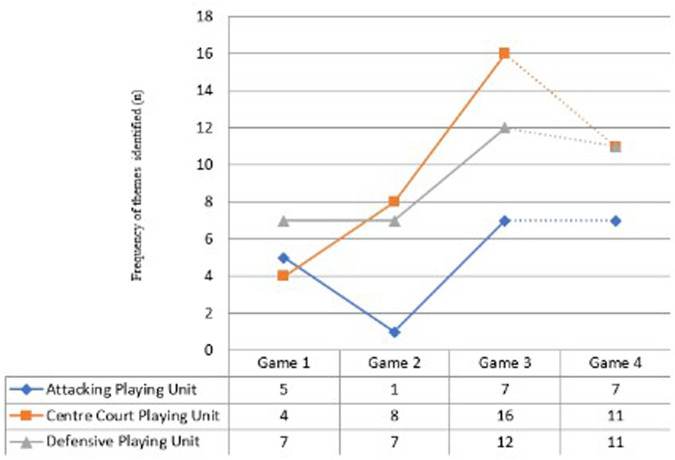
Illustrates the within game analysis for dominant theme “multiple players recognized and connected to own teammates” for all playing units (attack unit, centre court unit and defence unit) for games 1–4 for phase 1 of an ACP.

Figure 5 illustrates an increase in the identification of the theme relating to recognition of “*multiple players recognized and the connection to teammates*”. All playing units increased their identification of this theme over the four match period. Centre court players increase their identification progressively over games 1–3 with recognition values of *n* = 4, *n* = 8 and *n* = 16 respectively.

Figure 5 highlights a decline by all playing units in game 2. This replicates the finding of Bate and Richards (31), Richards et al. (4), and is perceived to be linked to players engaging in a “sensemaking” process where SMMs are being checked and refined. The remaining four themes have been identified as important but not dominant. These four themes are (i) “*SMM concept linked to space*”*;* (ii) “*area of the court and player positioning*” (e.g., offline/online); (iii*)* “*team SMMs*” (SMMs linked specifically to team philosophy); and (iv) “*teammates undefended*” (free player).

Defensive unit players remained consistent in their recognition of this theme over games 1 and 2, with a consistent value (*n* = 7) but accelerated rapidly in frequency of recall in games 3 (*n* = 12). Attacking unit players initially experienced a decrease from a recognition value of *n* = 5 in game 1 to *n* = 1 recall identification in game 2; with a rapid increase then observed to *n* = 7 identifications in game 3. All playing units maintained their identification rates following a two-week period of non-playing contact with identification values being higher than those first established in game 1. Such evidence suggests that players were identifying multiple teammates and their connection to each other more frequently as their playing experience (deliberate reflection) increased and SMM were refined, perhaps indicating the development of a collective perception of the playing context.

### Part 2: on-court, performance setting operationalising the SMMs

#### Analysis of on-court, “in-action” team performance

Analysis of performance indicators relating to training and international match data suggests that, over the eight-game period (4 × training plus 4  ×  international matches), ACP performance improved significantly. The justification in improved match statistics could be a response to an improvement in SMMs relating to the ACP, resulting in enhanced on-court player collaboration, shared situational awareness and collective team DM.

**Figure 6:**
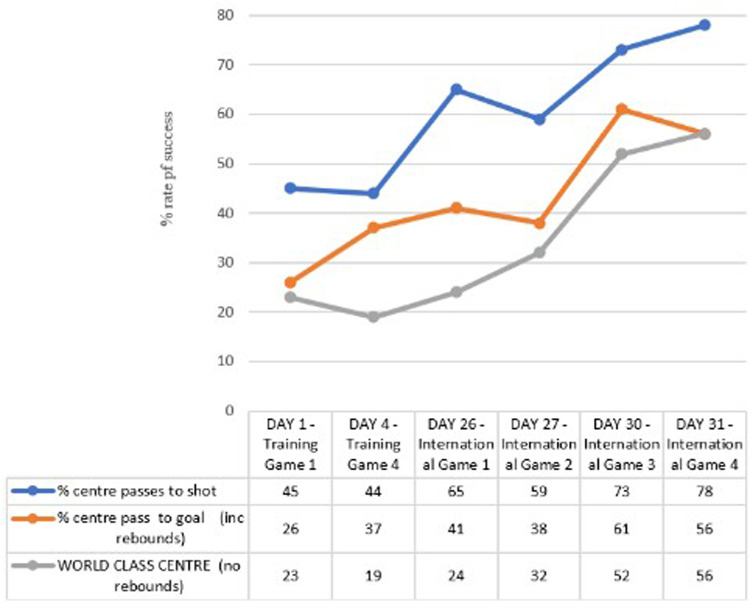
Time series to illustrate the development of connective play through the application of SMMs developed over the 31-day process. [Richards et al. (4), p. 9].

Figure 6 illustrates an increase in the frequency within which the ACP penetrated the attacking circle. Match statistics indicated a progression from 45% (Game 1, *n* = 17) in the first training match to 78% (Game 8, *n* = 39) for the fourth international game (a 31-day time period). The level of opposition in international matches was superior to training games, perhaps indicating the improvement in game 8 is underestimated and that the percentage for improvement might, in fact, be greater. Furthermore, not only did the frequency of the centre pass reaching the circle increase, but qualitative and quantitative data, highlights corresponding improvements in the quality of play, as observed over each phase of the play (Phases 1, 2 and 3). Using the “world class” criteria established for “centre pass to completions” (4), the number of “world class” centres recorded also increased from 23% (Game 1, *n* = 7) to 56% (Game 8, *n* = 25) for the final international match (Figure 6). Furthermore, inferential statistics using Mann–Whitney U (independent sample) demonstrated a significant difference between “world class” centre passes, and centre passes penetrating the circle when analysed as the number of centre passes performed during training camp (Game 1; Mdn = .94, *n* = 8) compared to the international stage (Game 8; Mdn = .66, *n* = 8) with values of *p* = 0.035 and *p* = 0.027 (*p* < .05) respectively.

## Discussion

The empirical findings indicate that team DM was enhanced when the DM Framework, employing CTA was integrated into off-court training within this team. Four key aspects have emerged from this study.

### Coaches’ SMMs

It is essential that, in the process of developing DM in teams, the initial vision of what performance will look like is perceived by the coach and their coaching team. Richards et al. (2), refer to this *alpha vision* as providing the foundations for building more complex layers of knowledge, which are essential to team DM. The *alpha* vision of performance is divided into a number of SMMs which informs and influences what information in the playing environment is attended to (36). The *alpha* vision includes both Psychomotor (Technical component of SMMs, information) and Psychosocial aspects (Non-technical component of SMMs; e.g., culture, politics, values etc; see (1). Only when this *alpha vision* has been clarified can it then be broken down into “chunks” and presented to the players. This is demonstrated in this study, with the division of the ACP into phases 1, 2 and 3 and the improvement in team DM.

### Developing team decision making

Empowering the players to contribute to discussions relating to the *alpha* vision of the ACP resulted in the design and development of SMMs. This created a shared common language amongst coaches and the players. The establishment of this new *beta* vision of performance (players and coaches shared vision) resulted in the identification of dominant factors and key determinants relating to the ACP. Further investigation is needed to explore the dominant factors and key determinants. Key determinants appear to be significant across all phases of an action (in this context phase 1, 2 & 3), with some factors being bespoke to a specific phase (dominant factors). Within context of this study, two dominant factors were influential in accelerating DM in an ACP for phase 1. These three factors were “*tactical team concepts specific to the playing philosophy of the team*”, “*recognition of multiple players*” and “*connection of movements between teammates and opposition*”. Consideration also needs to be given to two additional components, which although not recognized unanimously by players and coach did provide strong evidence to suggest that further investigation is required. These two additional components were: “*area of court*” (identified by players only) and “*numerical superiority*” (identified by coaches only). Both aspects relating to Shared situational awareness (6). It is, therefore not surprising that analysis indicated that players and coaches produced increased compatibility in SMMs, relating to the ACP, following the engagement with the DM Framework with CTA embedded. The compatibility of SMMs increased as the number of games progressed. The main distinguishing factor between players’ and coaches’ SMMs related to the enhanced complexity of coaches’ SMMs, understandable given the years gained in both playing and coaching at an elite performance level.

### Team decision-making (coach & player collaboration)

The ability of the team to incorporate reflection “*on–practice*” at an individual, unit and team level in a slow, deliberate, learning environment using video and CTA, allowed SMMs to be designed and refined. Such SMMs which had been developed off-court were transferred to the on-court, competitive setting. Additionally, the engagement with reflection in the off-court environment provided the mechanism to increase individual players’, units and the team's understanding of team tactical concepts. The reinforcement of reflecting on individual, unit and team performance collectively through TAPS resulted in the development of more complex playing concepts and exposed the players to new experiences. It appears that Klein et al.'s (37) proposal of the RPD and sensemaking theories make a valuable contribution in enhancing our understanding of how netball players developed SMMs. Klein's (24) cue-based theory of RPD, which incorporates both intuition and analysis of the situations, provides a vehicle for understanding how information in the performance setting is recognized, as players attend to information which is perceived to be of value in the context of the team's playing philosophy (2). However, the attendance to such information, while still acknowledging team playing philosophy, requires the perceived information to be contextualized in relation to the playing environment. As such, the team develops shared SA, where team members collectively identify a preferred (or dominant) action, based on the interpretation of data points in the context of the team's overall playing philosophy (1, 36). It is, therefore of value to consider the theories of RPD, SA and sensemaking in combination, to inform what information is being attended to, how it is being interpreted, and more importantly organized in context of unique parameters of each team. Sensemaking, therefore, has an emergent contribution to make to accelerated DM as a theory (16), as it progresses beyond merely understanding the information and addresses the connection between information which is identified as significant.

### On court team performance

The analysis of match data in the format of performance indicators, illustrated a statistically significant improvement in match performance over the period of the eight games (31-days). Given the elite nature of the team, and that physical and technical conditioning is already at its highest, the influencing fact was the integration of the reflective DM Framework with CTA. The accumulated evidence therefore suggests that team SMMs relating specifically to an ACP and developed off-court, were translated to enhance on-court in-action team DM. CTA is therefore presented as a method of facilitating a learning framework, through reflection to develop SMMs and tactical understanding.

In evaluating the study the following aspects are considered. The DM Framework proposed in this paper overcomes some of the limitations presented in other DM models, as both psychomotor and psychosocial elements are presented in an interconnected framework rather than in isolation. It has been acknowledged by academics (5) that practitioners need a greater understanding as to what factors are responsible for DM and therefore influence what information the players perceive and attend to, while playing in the competitive setting. This paper, therefore overcomes previous limitations and adds to the body of knowledge in the area of accelerating team DM by considering not only the psychomotor factors (e.g., visual and metacognitive processes) that determine DM in a team context, but also recognising and including psychosocial components of DM (e.g., pedagogy, empowerment, team philosophy, culture, and maturity). As the physiological level of the performers within this study are of the highest standard in context of physiological conditioning and technical ability, the enhancement in performance can only be through the design and operationalising of the team's unique SMM, empirically supporting the DM Framework. However, a limitation of this study is the recognition that the DM Framework presented within this paper has only been empirically tested in an elite context. To date the framework has mainly been used in elite senior sport (hockey, (2), Football, (31) [Olympic teams, (7, 38)], but has only been applied in a limited number of youth contexts [junior football, (10, 32)]. A solution to overcoming this limitation would be to consider the application of the DM Framework in both novice and sub-elite performance environments. Extending the model across the talent pathway from grass route to elite would help enhance our understanding of how decision-making and SMM change across the levels of expertise. A second limitation is to consider the application of the DM framework from a pedagogical perspective. Although previous work relating to the framework has explored it from a pedagogical delivery perspective (4) outlining the stages of delivery of the framework for coaches, more research is required to examine the DM framework from a coach education and training perspective. With further consideration given to its application in individual sports. Early work focusing on training decision-making from a pedagogical perspective in elite canoeing and dance has proved promising, see Simons and Richards (25, 39), and Wilson and Richards (40), respectively. Finally, the model has been extended and applied to military settings (8); Daden et al., 2023, (41); and is currently being explored in policing and fire rescue, demonstrating the application and transferability across domains.

### Summary and conclusion

The evidence presented in this paper indicates that the deliberate introduction of the Decision Making Framework (1), which provided a structured approach to embedding real-time reflection (hence the development of MM and team SMM) through CTA methods, enhanced the operationalisation of team tactical play in elite contexts. The framework which embeds real-time CTA approaches into the framework (during team meetings and after action reviews), enabled elite performers to accelerate the development of tactical SMMs relating to the ACP. In turn enhancing tactical team decision-making, when performed under competitive pressure.

The evidence presented in this paper indicates that the introduction of a slow, deliberate, off-court learning environment in the format of structured reflections in after action reviews, enabled elite netball players to develop and operationalise SMMs relating to the ACP. The development of the specific SMMs relating to an attacking centre pass enabled players to agree on what information contained within SMMs was significant, which enabled all individuals to perceive, prioritise and attend to information in the same way. The layering of information in a progressive manner through the Decision Making Framework, enabled players to comprehend a range of playing situations relating to variations of the attacking centre pass. The layering of cognitive information enabled players to increase their understanding of their own role, the role of teammates, and specific phases of the centre pass, and then contextualise this information in the larger context of other key factors such as the style of the opposition, the phase of the games and key situational factors. The progressive layering of cognitive information enabled the complexity of SMMs to be developed over the games, as players reflected and learnt from their on-court playing experience.

It is recognised that the evidence produced in this paper provides further verification to support the utilisation of the DM Framework (1) as a mechanism for developing high pressurised naturalistic team decision-making in complex settings. However, it is acknowledged that research needs to be continued for it to be fully used as a vehicle to accelerate team DM within wider naturalistic settings.

Engagement in this research has identified several future research directions. One direction of future work would focus on the application of the framework, under different team parameters (e.g., age, experience, expertise) and decision contexts (performance settings) and across multiple domains. These include the application across other domains, e.g., emergency services, biosector and military. In addition, the nature of the task, considering team numbers, time context and level of task complexity and duration are factors which still need to be explored. Secondly, the findings from this paper have provoked interest in the exploration and refinement of some of the mechanisms located within the framework—specifically stages relating to Model 1 and 2. Finally, as highlighted above, interest has developed into the exploration of CTA methods, in addition to developing a greater understanding of the classification of team cognitions in sports.

To conclude, the development of the specific SMMs relating to an ACP, enabled players to agree on what information contained within SMMs was significant, which enabled all individuals to perceive, prioritise and attend to information in the same way. The layering of information in a progressive manner (DM Framework) enabled players to comprehend a range of playing situations relating to variations of the ACP, and in turn co-ordinate their actions. Using the DM Framework, the findings have illustrated that players, when empowered to construct SMMs, develop SMMs that are also closely aligned with fellow teammates. The creation of such SMMs in a slow, deliberate learning context provided the framework that could be used to guide and shape players’ cognition in complex and dynamic naturalistic performance settings. The findings of this research has wider implication for multiple domains, where teams function in a naturalistic setting, which are dynamic and continually changing, and which requires teams to be agile and adaptive in their collective response. It is appreciated that a significant amount of work is still required if team DM is to be fully understood within wider contexts.

## Data Availability

Data can be made available on request to the corresponding author.
